# Comparison of oral versus parenteral methotrexate in the treatment of rheumatoid arthritis: A meta-analysis

**DOI:** 10.1371/journal.pone.0221823

**Published:** 2019-09-06

**Authors:** Andreea M. Bujor, Sahar Janjua, Michael P. LaValley, Josefina Duran, Jürgen Braun, David T. Felson

**Affiliations:** 1 Department of Medicine, Boston University School of Medicine, Boston, Massachusetts, United States of America; 2 School of Public Health, Boston University, Boston, Massachusetts, United States of America; 3 Department of Clinical Immunology and Rheumatology, Pontificia Universidad Católica de Chile, Satinago, Chile; 4 Institut für angewandte Statistik Dr. Jörg Schnitker GmbH, Bielefeld, Germany; Keio University, JAPAN

## Abstract

**Objective:**

Studies suggest that parenteral MTX may be more efficacious than the oral form at equivalent doses for the treatment of rheumatoid arthritis. We carried out a meta-analysis to compare the efficacy of oral versus parenteral MTX in RA.

**Methods:**

PubMed, Web of Science and Embase were systematically searched from inception to June 8^th^ 2017 and reviewed following PRISMA 2009 guidelines, by two independent reviewers. To be included, trials had to study adults with RA randomized to the same dose of either oral or parenteral MTX. The primary endpoint was ACR20 at 6 months. Intention-to-treat analysis results were used when possible. Data from direct comparisons between oral and parenteral methotrexate quantitatively analyzed using maximum likelihood random effects meta-analysis. Relative treatment effects were generated as an odds ratio [OR] (OR>1 indicated a benefit for parenteral therapy).

**Results:**

The search yielded 357 papers or abstracts. After review of titles or abstracts and full text papers, we found 4 that met inclusion criteria with 703 patients randomized. Dose of MTX started at 15mg/week and increased up to 25mg/week. The summary OR for achieving ACR20 using parenteral vs. oral MTX was 3.02 (95% CI 1.41, 6.46), with no significant difference in the risk for all adverse events.

**Conclusion:**

Parenteral MTX therapy had significantly higher odds than oral MTX of achieving reduction in disease activity. We propose that parenteral MTX is more effective than weekly oral MTX; its widespread use may lead to better control of disease and a decrease in demand for biologic agents.

## Introduction

Rheumatoid arthritis is a chronic autoimmune inflammatory disease, with an estimated annual incidence of 50 per 100,000 and prevalence of approximately 0.8% [1] [2] [3], that results in a significant socioeconomic burden. Early recognition of the disease, followed by timely optimal management within the so called “window of opportunity”, leads to less joint damage, less disability and overall mortality, as well as more frequent disease remissions.

Management of RA has changed significantly over the years, as breakthroughs in the understanding of disease pathogenesis have led to a tremendous growth in the number of available treatments. ACR and EULAR updated their RA management guidelines to provide similar treatment algorithms, both recommending Methotrexate monotherapy as the first strategy for early RA [4] [5]. EULAR and ACR recommendations are to increase the Methotrexate dose until disease control has been achieved, and up to 25 mg daily. These practice guidelines also recommend considering other conventional DMARDs or changing to biologics if response to MTX is suboptimal.

Numerous RCT compared oral methotrexate to biologics in early RA. While combination therapy may be more effective than monotherapy in methotrexate naïve patients, [6] [7] [8], a recent meta-analysis reported that, in Methotrexate naïve patients with early RA, monotherapy with TNF inhibitors was not more efficacious than MTX alone for any of the outcomes studied [9]. Furthermore, Methotrexate has a more favorable cost and risk to benefit ratio, thus remaining the recommended drug of choice for initial treatment of RA.

In a recent systematic review, we suggested that the results of clinical trials comparing Methotrexate with biologics for the treatment of RA are likely biased towards showing a greater efficacy for the later, due to the use of a suboptimal MTX dose and route of administration in these trials [10].

A dose-dependent efficacy and toxicity for methotrexate in the treatment of RA has also been demonstrated [11] [12] [13]. While dose optimization is recognized as an important aspect of MTX monotherapy, less emphasis has been made on the optimal route of administration. Pharmacokinetic analyses have shown that the bioavailability of Methotrexate increases with parenteral administration, and this is particularly true for doses higher than 15mg/day [14]. Several studies have estimated that the bioavailability of oral methotrexate is approximately one third lower than its parenteral formulation, and that the route of administration also influences its safety and tolerability [15]. Evidence also suggests that parenteral MTX may have greater efficacy in RA management compared to oral MTX, both in patients with early [16] [13] and longstanding disease [17]. However, the evidence supporting the superiority of parenteral vs. oral MTX is far from definitive. The one large randomized trial directly examining this issue [16] allowed for switching of patients from one to another preparation prior to analysis of outcomes and therefore did not follow the intent to treat principle. Another study by Hazlewood looked at the initial treatment with subcutaneous versus oral MTX in patients with early RA, and found that the parenteral form was associated with lower average DAS-28 scores, lower treatment discontinuations, and no difference in toxicity compared to the oral formulation. While this large study supports the notion of increased efficacy of the parenteral formulation, we did not include it in our analysis, as it was an observational study that also used slightly higher doses in the parenteral group, thus making it difficult to establish definite causation [13]. The rest of the available data is mostly from observational studies with biases in allocation and no details on study methods.

Given the important impact that route of administration may have on the bioavailability and efficacy of methotrexate, its beneficial cost and side effect profile compared with biologics, along with the lack of rigorous data, the aim of our meta-analysis was to assemble, after comprehensive search, the randomized trial data directly comparing the same dose of oral vs. parenteral methotrexate, to compare their efficacy and safety.

## Materials and methods

### Inclusion criteria

We included randomized trials of 24 weeks’ duration that compared oral versus parenteral methotrexate. We included studies of adults (age >18 years) with rheumatoid arthritis, according to 1958, 1987, or 2010 classification criteria. We applied no dose restriction to methotrexate, but the doses of oral vs. parenteral must be the same. Outcomes must include a composite symptom/sign outcome including ACR20, SDAI, CDAI or DAS.

### Search strategy

We identified randomized trials including abstracts by searching databases including PubMed, Web of Science and Embase, from their inception to June 8^th^ 2017. The search strategies contained subject headings and keywords for “rheumatoid arthritis”, “methotrexate”, “oral” “subcutaneous”, “parenteral”, “injectable”, and “intramuscular”. Bibliographies of review articles and other selected articles were also independently reviewed, and additional trials were included by manual search. Two review authors (AB, SJ) independently screened articles for inclusion by title or abstract and full text when necessary. Any disagreement between them was resolved by consensus or discussion with a third review author (DF).

### Data extraction

We extracted the following data from the included articles: date of publication, country of origin, number of participants in each group, demographic data of participants including age and gender, dosages of drugs administered for each group, all endpoints available in every study including DAS28, SDAI-LDA, ACR20, ACR50, ACR70, with primary endpoint being ACR 20. We also extracted data on side effects due to adverse events and serious adverse events. Data extraction was done by two independent reviewers and disagreements were resolved by consensus.

### Statistical analysis

We attempted to create one uniform outcome for all studies, the ACR20. One of the studies allowed treatment switching (Braun) before the outcomes were assessed at 24 weeks. We contacted the authors and they reanalyzed the data at the 16 week time point, just before treatment switching, so as to preserve data on randomized treatment for the primary outcome of ACR20. They were unable to carry out analyses of other RA outcomes. In the trial by Dhaon et al, we reanalyzed data to provide ACR20 values by using a statistical crosswalking strategy to calculate ACR20 data from SDAI-LDA scores, since ACR data was not directly available. Data from the Exxelerate trial [18] (which provided rates of both ACR20 and SDAI-LDA in treated groups and permitted this comparison) were used. Intention-to-treat analyses were used when possible. Data from direct comparisons between oral and parenteral methotrexate were pooled and quantitatively analyzed using maximum likelihood random effects meta-analysis. Relative treatment effects were generated as odds ratio [OR] (OR>1 indicated a benefit for parenteral therapy). We also examined the mean difference in ACR20 rates between parenteral and oral MTX. The Higgins *I*^2^-test was used to explore the extent of heterogeneity across the included articles [19]. I^2^ values of 25, 50 and 75% correspond to low, medium and high levels of heterogeneity, respectively.

### Quality analysis

The quality of methods from the included trials was measured independently by two reviewers. Risk of bias assessment was done using Cochrane methodology [20] by evaluating for random sequence generation, allocation concealment, incomplete or selective outcome data reporting, blinding of participants and personnel, blinding of outcome assessment, attrition, and other sources of bias. For each domain, the risk of bias was assessed as either low, high or undetermined. An Egger test was also performed to assess publication bias.

## Results

### Search results

**Fig 1:**
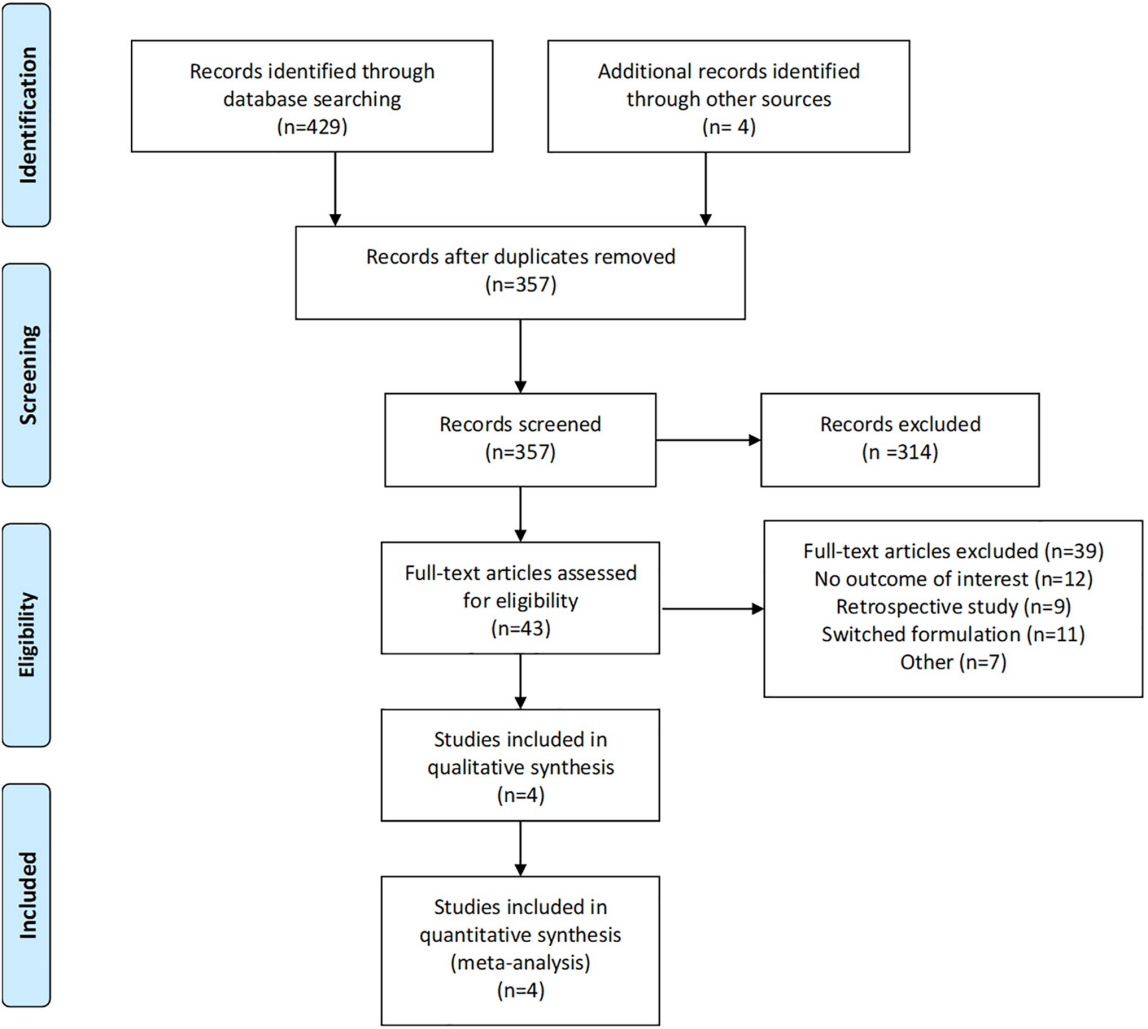
Flow chart of study selection.

The initial electronic search yielded 429 papers or abstracts. Additional manual search using bibliography of select articles identified 4 more records. After removal of duplicates, 357 papers or abstracts remained. The titles or abstracts of these records were then reviewed, and additional 314 records were further excluded. The remaining 43 full-text papers or abstracts were examined, and we found 4 that met the inclusion criteria. These trials compared the clinical efficacy, tolerability and safety of oral vs parenteral Methotrexate, and were included in our analysis. Fig 1 shows the flow chart of study selection.

### Characteristics of included studies

S1 Table shows a summary of the characteristics of each included study. Among these four included trials, there were a total of 695 patients randomized, with 370 in the oral group, and 325 in the parenteral group. One of the trials had three arms, two of which were oral treatments at the same cumulative doses, but in one arm the MTX was given in split doses [21]. To be more accurate in our assessment, we included only the group of patients that took MTX as a single dose weekly, similar to the patients in all other three studies. Although no significant differences in disease duration were recorded between the oral and parenteral groups in each individual study, the trial by Braun included mostly patients with early RA, with a median disease duration of 2.1 and 2.5 months respectively, as opposed to the studies by Dhaon [21] and Islam [22], were patients had a disease duration of 4–6 years on average. One of the studies was an abstract (Ahmed) and did not provide data on disease duration or patient’s age [23]. The mean/median age of the patients did not differ significantly between the two groups in any of the remaining three studies, ranging between 40 and 59 years.

The parenteral route was subcutaneous for three studies, and intramuscular for one. Dose of MTX started at 15 mg/week and increased to as high as 25 mg/week. Although different between individual studies, the analyzed studies used identical oral and parenteral doses at any given time in their protocol, except for the Braun trial which allowed switching for poor response at 16 weeks and included only 24 week data. The authors of that study were contacted and they reanalyzed data at 16 weeks, and the new results were included in this meta-analysis.

### Risk of bias

After independent evaluation by two different reviewers, and discussion of any disagreements, the results for the quality assessment were generated as a table, and risk of bias was evaluated by Egger’s test. A major limitation of the studies was the lack of available information regarding the study methods. Three of the studies generated a random sequence, and while the fourth study also randomly divided patients in groups, it did not offer details regarding the method of randomization. Only one study used concealed allocation, and blinded outcome assessment. The rest of the studies had less clear methods. S2 Table summarizes the results for the risk of bias assessment. Egger’s test was not statistically significant, with a calculated p value of 0.27.

### Results of meta-analyses

#### Efficacy outcome

The primary endpoint in all but one trial (Dhaon) was ACR20 at 24 weeks. Data from Dhaon trial was reanalyzed as described in Materials and Methods to provide the necessary endpoints included in our study. The revised data form the Dhaon study showed that in the oral MTX group there were 21 ACR20 responders from a total of 45 patients, and in the parenteral group 28 responders from a total of 45 patients. This resulted in a risk difference in ACR20 of 0.16, with a 95% CI (-0.05, 0.34), and an odds ratio of 1.88, with a 95% CI (0.81, 4.36), when comparing parenteral to oral MTX.

**Fig 2:**
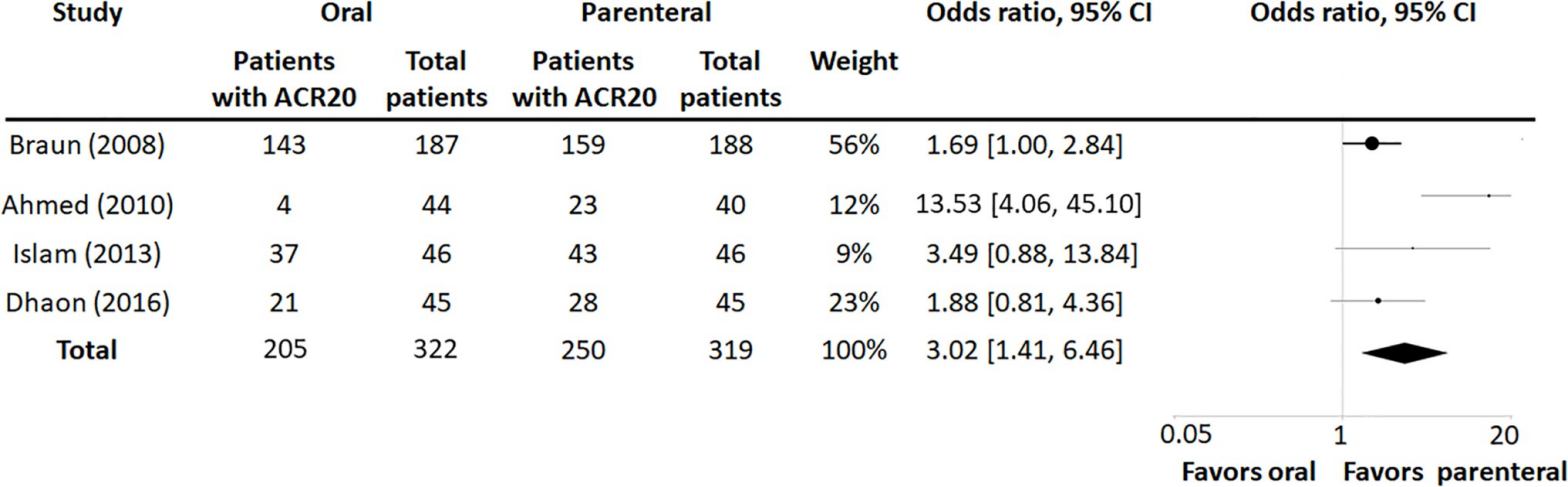
Summary OR for achieving ACR20 using parenteral vs. oral MTX.

In each trial, ACR20 rates were higher for those randomized to parenteral than to oral MTX. The individual and combined estimates for random effects across the 4 included studies are shown in Fig 2, with the odds ratio for achieving ACR20 using parenteral vs. oral MTX calculated at 3.02 (95% CI 1.41, 6.46), with an I^2^ measure of heterogeneity of 70.07%.

Those on parenteral Methotrexate had a 20.2% increased risk of attaining ACR20 improvement (95% CI 5.0%, 35.3%) compared to those on oral MTX, with an I^2^ measure of heterogeneity of 82%.

#### Adverse events

**Fig 3:**
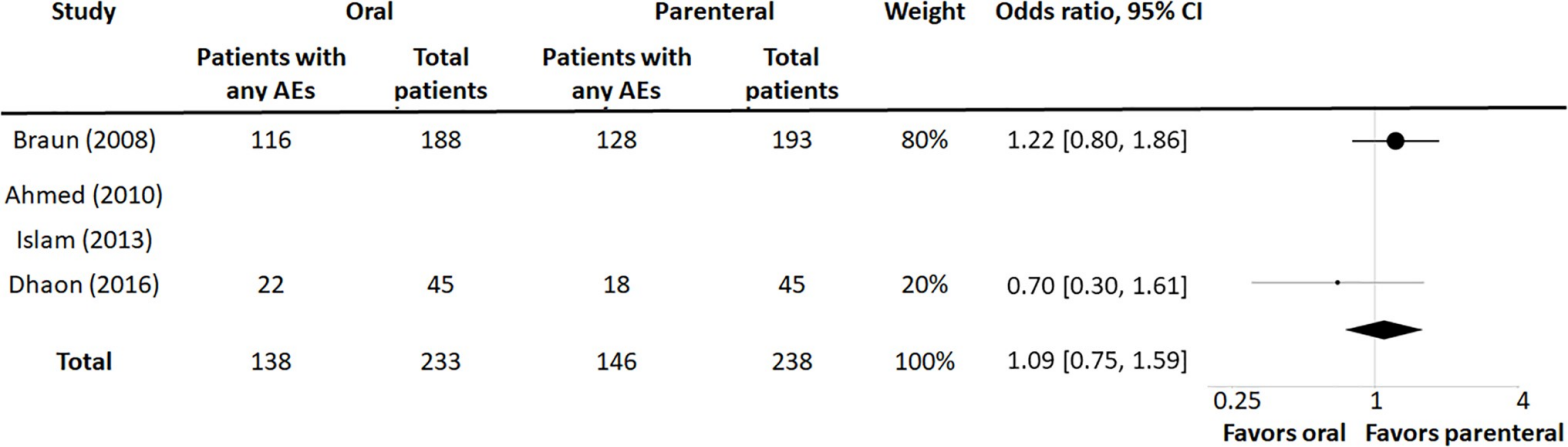
Summary OR for adverse effects data (any AE).

**Fig 4:**
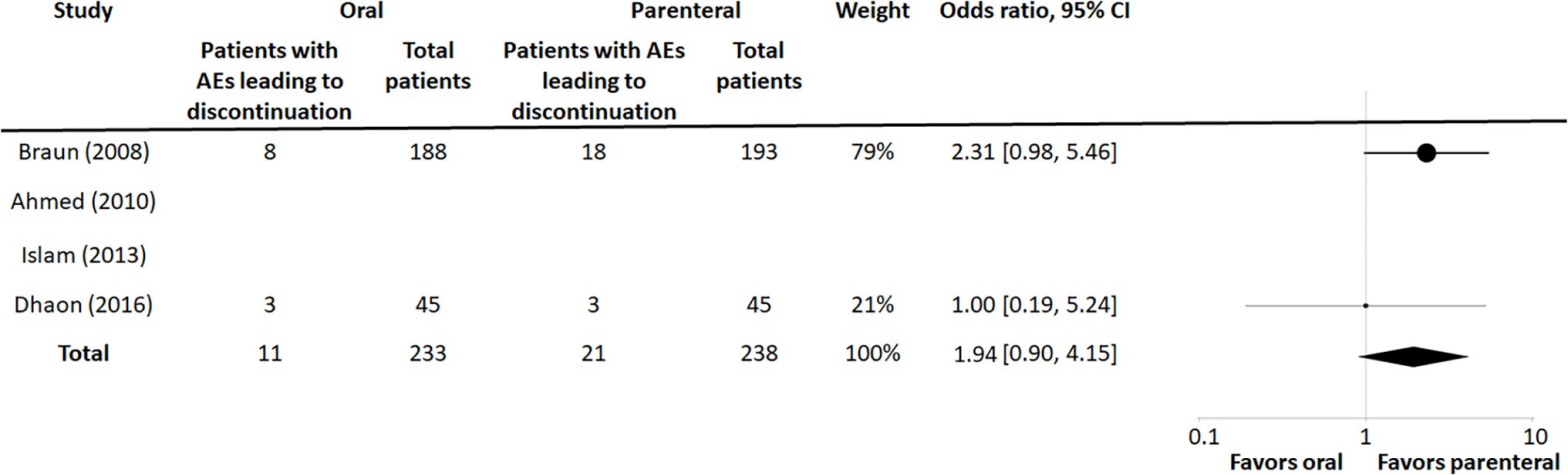
Summary OR for adverse effects data.

Of the four trials included in this meta-analysis, three reported adverse events. Two of these studies reported adverse events using similar categories, including adverse events leading to discontinuation. Further analysis of these two trials showed no difference in any adverse events between groups, with an OR of 1.09 (95% CI 0.75, 1.59, p = 0.82) (Fig 3). The OR for adverse events leading to discontinuation was slightly but not significantly increased in the parenteral group, with OR 1.94 (95% CI 0.90, 4.15, p = 0.1) (Fig 4).

## Discussion

Methotrexate is the cornerstone therapy for early RA, and is frequently used as a comparator drug in clinical trials with biologics in RA. However, the impact of route of administration on its efficacy has been largely overlooked. Common practice is to start with a lower dose, i.e. 15 mg weekly orally (po), and then to gradually increase to a maximum of 20 or even 25 mg weekly po, based on clinical response. If disease control is not achieved with this dose, many clinicians switch to a different drug, with TNF inhibitors being usually favored. As previously mentioned, in a recent meta-analysis there was no significant difference between Methotrexate and TNF biologic monotherapy in Methotrexate naïve RA patients. Since bioavailability of methotrexate is higher with parenteral formulations, without an increase in adverse effects, it is important to understand whether this method of delivery also has greater efficacy. Several observational studies and few randomized control trials have suggested that this might be the case, but these trials were poorly controlled and mostly done in countries with lower socioeconomic status, where Methotrexate might be more appealing due to its cost and lower risk for reactivation of latent infections.

While this is not the first meta-analysis of oral versus parenteral Methotrexate in RA, our study brings important contributions to the existing supporting evidence that favors greater efficacy for the parenteral formulation. There was a single additional study that performed a meta-analysis of this kind, but the authors only included two trials to calculate the outcomes of clinical efficacy, namely the Braun and Islam studies [24]. While they also reached the same conclusion, our study was more comprehensive and probably used more valid data. Firstly, the Braun study allowed for switching of patients from oral to the parenteral preparation, or to increase the dose for the later at 16 weeks, if they did not meet the ACR20. The meta-analysis by Li et al reported the data at 24 weeks for this particular trial. Given the Braun study is the largest and best controlled randomized clinical trial published to date, in order to eliminate the risk of potential bias, we reached out to the authors and requested that they reanalyze the data for the primary outcome of ACR20 at the 16-week time point, just before treatment switching. The reanalyzed data was then included in our final analysis and it confirmed the superiority of the parenteral formulation.

Our meta-analysis also includes two additional studies not included in the previously published meta-analysis, both supporting the previous findings. The study by Dhaon, the second largest randomized control trial, postdated the Li et al meta-analysis.

Regarding the adverse effects, we found no significant difference in the risk for all adverse effects, which is in contradiction to what was previously published. However, the data regarding the side effects in these trials was incomplete, and further studies are required to reach a more definitive conclusion.

Our study has several limitations. Firstly, out meta-analysis included only 4 trials, due to lack of other available, published randomized control trials. Secondly, we included a trial reported as abstract in this analysis, the Ahmed trial. Although absence of peer review has been associated with lower quality scores, we chose to include this trial due to the very limited number of randomized control trials published looking at the current topic. This study provided very little additional information besides the ACR20, and no details regarding the rate of adverse effects in the two groups (except that they were similar), thus was not included in the final adverse effects analysis.

Another potential limitation is that while the Dhaon study used an intention to treat analysis, it provided only SDAI scores for primary endpoint and thus required further statistical analysis to extrapolate the results to ACR20, in order to be accurate in our assessment. Our study did not evaluate the potential benefit that may come with split oral MTX dose, presumably due to increased MTX bioavailablity compared to weekly dosing [25]. The trial by Dhaon showed greater efficacy for the oral split versus weekly oral MTX [21], in diasgreement with a previous trial that showed no difference [26]. This could be attributed to dosing differences between the two studies, with Dahon study using higher MTX doses. It is possible that the benefit that we found with parenteral MTX is similarly due to higher bioavailability, and that this therapeutic advantage may also be achieved with splitting the oral dose, as seen in the Dahon study. Prospective randomized controlled trials are required to confirm these findings before any changes to therapy with MTX could be justified.

While each individual trial used comparable doses between the two arms, the doses used across these four trials were not uniform. Given the fact that systemic exposure of oral MTX plateaus at doses above 15 mg/week, those variations may be important [27]. Nevertheless, this study still supports the notion that parenteral Methotrexate is more effective than its oral formulation.

An additional limitation is that besides the Braun study, which included only MTX-naïve patients with early disease (under 3 months), two studies included patients that were already on oral methotrexate and had much longer disease duration. While the study by Ahmed et al included only MTX-naïve patients, the abstract did not provide any details about disease duration, and authors did not respond to our request for additional information.

Lastly, there was significant heterogeneity among the included studies, with most dissimilar results stemming from the Ahmed trial. The high heterogeneity observed could be due to variation in study design and/or population. Given the small number of studies, we did not attempt to reduce and interpret the heterogeneity in subgroup analyses. To address this shortcoming, we used random effects model to assess for outcomes.

## Conclusion

In conclusion, despite several limitations, our meta-analysis demonstrated that parenteral MTX therapy has significantly higher odds than single dose weekly oral MTX of achieving reduction in disease activity, with no increased adverse effects.

## Supporting information

S1 Table Study characteristics. Table showing the characteristics of the studies included in the meta-analysis.
(TIF)

S2 Table Risk of bias. Details on the methodological quality of the included trials (l-low, h-high, ?-undetermined).
(TIF)

S3 Table PRISMA checklist. Details regarding adherence of the meta-analysis to PRISMA checklist.
(DOC)

S1 Fig Embase search criteria. Criteria used to search Embase.
(PDF)

S2 Fig Web of Science search criteria. Criteria used to search Web of Science.
(PDF)
