# Supplementary figures and images for: Comparison of oral versus parenteral methotrexate in the treatment of rheumatoid arthritis: A meta-analysis
Source: PLoS One. 2019 Sep 6;14(9):e0221823. doi: 10.1371/journal.pone.0221823 (PMC6731021; doi:10.1371/journal.pone.0221823)

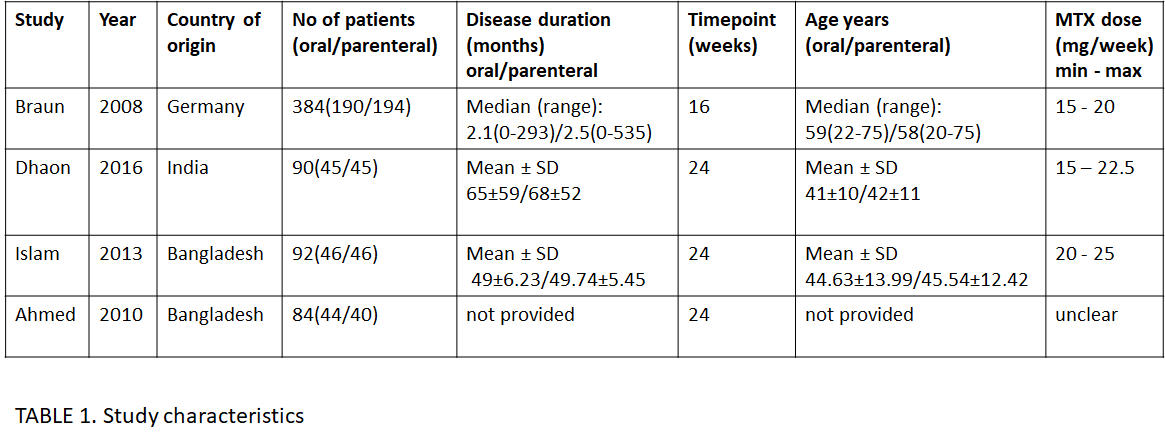

Supplement: S1 Table — Table showing the characteristics of the studies included in the meta-analysis. (TIF) [file pone.0221823.s001.tif]

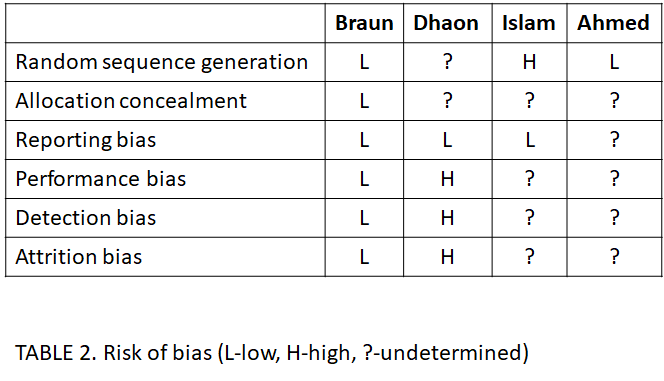

Supplement: S2 Table — Details on the methodological quality of the included trials (l-low, h-high, ?-undetermined). (TIF) [file pone.0221823.s002.tif]
