# Supplementary material for: Comparison of oral versus parenteral methotrexate in the treatment of rheumatoid arthritis: A meta-analysis
Source: PLoS One. 2019 Sep 6;14(9):e0221823. doi: 10.1371/journal.pone.0221823 (PMC6731021; doi:10.1371/journal.pone.0221823)
Supplement: S1 Fig — Criteria used to search Embase. (PDF) [file pone.0221823.s004.pdf]

Embase Session Results

No.	Query	Results
#6	#1 AND #5	2,191
#5	#2 OR #3 OR #4	1,388,654
#4	'injection'/exp OR injection	616,949
#3	'parenteral drug administration'/exp OR parenteral AND drug AND administration	773,925
#2	‘subcutaneous drug administration'/exp OR subcutaneous AND drug AND administration	244,674
#1	'rheumatoid arthritis'/exp OR 'arthritis deformans' OR 'arthritis, rheumatoid' OR 'arthrosis deformans' OR 'beauvais disease' OR 'chronic polyarthritis' OR 'chronic progressive poly arthritis' OR 'chronic progressive polyarthritis' OR 'chronic rheumatoid arthritis' OR 'disease, beauvais' OR 'infantile rheumatoid arthritis' OR 'inflammatory arthritis' OR 'polyarthritis, primary chronic' OR 'primary chronic polyarthritis' OR 'progressive polyarthritis, chronic' OR 'rheumarthritis' OR 'rheumatic arthritis' OR 'rheumatic polyarthritis' OR 'rheumatism, chronic articular' OR 'rheumatoid arthritis' AND ('methotrexate'/exp OR '4 amino 10 methylfolic acid' OR '4 amino 10 methylpteroylglutamic acid' OR '4 amino n10 methylpteroylglutamic acid' OR 'mtx' OR 'a methopterine' OR 'abitrexate' OR 'amethopterin' OR 'amethopterine' OR 'ametopterine' OR 'antifolan' OR 'biotrexate' OR 'canceren' OR 'cl 14377' OR 'cl14377' OR 'emtexate' OR 'emthexat' OR 'emthexate' OR 'emtrexate' OR 'enthexate' OR 'farmitrexat' OR 'farmitrexate' OR 'farmotrex' OR 'folex' OR 'folex pfs' OR 'ifamet' OR 'imeth' OR 'intradose mtx' OR 'lantarel' OR 'ledertrexate' OR 'maxtrex' OR 'metex' OR 'methoblastin' OR 'methohexate' OR 'methotrate' OR 'methotrexat' OR 'methotrexat ebewe' OR 'methotrexate' OR 'methotrexate lpf' OR 'methotrexate preservative free' OR 'methotrexate sodium' OR 'methotrexate sodium preservative free' OR 'methotrexato' OR 'methoxtrexate' OR 'methrotrexate' OR 'methylaminopterin' OR 'methylaminopterine' OR 'meticil' OR 'metoject' OR 'metothrexate' OR 'metothrexate sodium' OR 'metotrexat' OR 'metotrexate' OR 'metotrexin' OR 'metrex' OR 'mexate' OR 'mexate-aq' OR 'mexate-aq preserved' OR 'mpi 5004' OR 'mpi5004' OR 'n [4 [(2, 4 diamino 6 pteridylmethyl) methylamino] benzoyl] glutamic acid' OR 'neotrexate' OR 'nordimet' OR 'novatrex' OR 'nsc 740' OR 'nsc740' OR 'otrexup' OR 'rasuvo' OR 'reumatrex' OR 'rheumatrex' OR 'rheumatrex dose pack' OR 'sodium methotrexate' OR 'texate' OR 'texate-t' OR 'texorate' OR 'trexall' OR 'xaken' OR 'zexate') AND ('oral drug administration'/exp OR 'administration, oral' OR 'drug administration, oral' OR 'oral administration' OR 'oral drug administration' OR 'oral drug intake' OR 'p.o. administration' OR 'p.o. dosage' OR 'p.o. dose' OR 'p.o. drug administration' OR 'p.o. drug intake' OR 'per os drug administration')	5,924
