# Supplementary material for: Comparison of oral versus parenteral methotrexate in the treatment of rheumatoid arthritis: A meta-analysis
Source: PLoS One. 2019 Sep 6;14(9):e0221823. doi: 10.1371/journal.pone.0221823 (PMC6731021; doi:10.1371/journal.pone.0221823)
Supplement: S2 Fig — Criteria used to search Web of Science. (PDF) [file pone.0221823.s005.pdf]

Web of Science

# 1	146,153	TS=(Rheumatoid NEAR/3 Arthriti*)
# 2	46,067	TS=(Methotrexate OR Amethopterin OR "Methotrexate, (D)-Isomer" OR "Methotrexate, (DL)-Isomer" OR Mexate OR "Methotrexate Sodium" OR "Sodium, Methotrexate" OR "Methotrexate, Sodium Salt" OR "Methotrexate, Disodium Salt" OR "Methotrexate Hydrate" OR "Hydrate, Methotrexate" OR "Methotrexate, Dicesium Salt" OR "Dicesium Salt Methotrexate")
# 3	53,003	TS=(Oral* NEAR/3 Administration)
# 4	692,414	TS=(Subcutaneous NEAR/3 Injection*) OR TS=(Parenteral NEAR/3 Infusion*) OR TS=(Intra-Abdominal OR Intra Abdominal NEAR/3 Infusion*) OR TS=(Peritoneal OR Interperitoneal NEAR/3 Infusion*) OR TS=(injection*)
# 5	18	#4 AND #3 AND #2 AND #1
# 6	109	#3 AND #2 AND #1
# 7	740,836	#4 OR #3
# 8	665	#7 AND #2 AND #1
# 9	16	#3 AND #2 AND #1 **Refined by: TOPIC:** (injection)
# 10	47	#3 AND #2 AND #1 **Refined by: TOPIC:** (subcutaneous or parenteral or injectable)
